# The effect of inbreeding rate on fitness, inbreeding depression and heterosis over a range of inbreeding coefficients

**DOI:** 10.1111/eva.12145

**Published:** 2014-02-07

**Authors:** Nina Pekkala, K Emily Knott, Janne S Kotiaho, Kari Nissinen, Mikael Puurtinen

**Affiliations:** 1 Department of Biological and Environmental Science, University of JyväskyläJyväskylä, Finland; 2 Natural History Museum, University of JyväskyläJyväskylä, Finland; 3 Finnish Institute for Educational Research, University of JyväskyläJyväskylä, Finland; 4 Centre of Excellence in Biological Interactions, University of JyväskyläJyväskylä, Finland

**Keywords:** genetic distance, genetic divergence, genetic drift, interpopulation hybridization, population size

## Abstract

Understanding the effects of inbreeding and genetic drift within populations and hybridization between genetically differentiated populations is important for many basic and applied questions in ecology and evolutionary biology. The magnitudes and even the directions of these effects can be influenced by various factors, especially by the current and historical population size (i.e. inbreeding rate). Using *Drosophila littoralis* as a model species, we studied the effect of inbreeding rate over a range of inbreeding levels on (i) mean fitness of a population (relative to that of an outbred control population), (ii) within-population inbreeding depression (reduction in fitness of offspring from inbred versus random mating within a population) and (iii) heterosis (increase in fitness of offspring from interpopulation versus within-population random mating). Inbreeding rate was manipulated by using three population sizes (2, 10 and 40), and fitness was measured as offspring survival and fecundity. Fast inbreeding (smaller effective population size) resulted in greater reduction in population mean fitness than slow inbreeding, when populations were compared over similar inbreeding coefficients. Correspondingly, populations with faster inbreeding expressed more heterosis upon interpopulation hybridization. Inbreeding depression within the populations did not have a clear relationship with either the rate or the level of inbreeding.

## Introduction

The effects of inbreeding, genetic drift and interpopulation hybridization on fitness are relevant for many basic and applied questions in ecology and evolutionary biology, such as metapopulation dynamics (Hanski 1999), evolution of mating and dispersal strategies (Pusey and Wolf 1996), speciation (Coyne and Orr 2004), success of invasive species (Ellstrand and Schierenbeck 2000) and conservation of endangered species (Hedrick et al. 2011). Inbreeding (mating between close relatives) increases offspring homozygosity and usually results in reduced fitness. In homozygous genotypes, recessive deleterious alleles are unmasked and benefits of heterozygosity in overdominant loci are lost (Charlesworth and Willis 2009). Genetic drift (random fluctuation in allele frequencies) may also depress fitness by causing deleterious alleles to accumulate and fix in the population (Lande 1994; Lynch et al. 1995a,b). Hybridization among genetically differentiated populations, on the other hand, is known to have the potential to alleviate the effects of inbreeding and drift by increasing heterozygosity in the population (Whitlock et al. 2000).

When population size is small, inbreeding and genetic drift both increase because the number of individuals contributing to each generation is limited (Keller and Waller 2002). Consequently, average fitness in a small population is expected to decrease from generation to generation as the level of inbreeding (i.e. homozygosity) increases (Crow and Kimura 1970; Wang et al. 1999; Keller and Waller 2002). Indeed, a positive relationship between population fitness and heterozygosity is often observed in experimental studies and in the wild (see e.g. Keller and Waller 2002; Reed and Frankham 2003; Spielman et al. 2004). As the average homozygosity in a population increases, the difference in homozygosity between offspring of close relatives and offspring from random matings decreases. Therefore, the so-called within-population inbreeding depression (i.e. the reduced fitness of offspring from inbred mating, when compared to offspring from random mating within the same population) is expected to decrease (Wang et al. 1999; Theodorou and Couvet 2006). Low within-population inbreeding depression is commonly observed in populations that have high average level of inbreeding (reviewed in Byers and Waller 1999).

The relationships between population inbreeding level and population mean fitness, and between population inbreeding level and within-population inbreeding depression, however, may not always be that simple. As recessive deleterious alleles become expressed with increasing homozygosity, selection can act to remove them from the population (Hedrick 1994; Glemin 2003). In theory, effective purging of deleterious alleles could restore population fitness to, or even above, the original level (Theodorou and Couvet 2006). Although empirical evidence for the effectiveness of purging is inconsistent (see e.g. Byers and Waller 1999; Crnokrak and Barrett 2002; Boakes et al. 2007; Leberg and Firmin 2008), some empirical studies do suggest that the relationship between population fitness and inbreeding level can be affected by purging selection (Reed et al. 2003; Larsen et al. 2011; Pekkala et al. 2012b).

The effectiveness of purging is expected to depend on inbreeding rate. Inbreeding rate refers to the rate at which homozygosity in a population increases: the smaller the population, the faster the increase in homozygosity from generation to generation (Falconer and Mackay 1996). With fast inbreeding, selection is expected to be efficient only against highly deleterious alleles, whereas with slow inbreeding also less harmful alleles can be under effective selection (Wang et al. 1999; Glemin 2003; Theodorou and Couvet 2006). This can have consequences on both population mean fitness and within-population inbreeding depression. When populations that experience either slow or fast inbreeding are compared at the same level of inbreeding, those with slow inbreeding are expected to show higher average fitness and lower within-population inbreeding depression because of more effective purging (Wang et al. 1999; Theodorou and Couvet 2006). At high levels of inbreeding, however, populations experiencing slow inbreeding may actually display higher within-population inbreeding depression because such populations are expected to be more heterozygous at loci under selection (Wang et al. 1999; Theodorou and Couvet 2006). Higher within-population inbreeding depression with slow inbreeding could also result from more efficient selection to maintain heterozygosity at overdominant loci (Kristensen et al. 2005; Demontis et al. 2009). Empirical evidence for the effect of inbreeding rate on within-population inbreeding depression is still lacking. Furthermore, empirical studies on the effect of inbreeding rate on fitness do not always support the prediction of more effective selection with slow inbreeding (Mikkelsen et al. 2010; Kristensen et al. 2011). Also, although several studies have examined the effect of inbreeding rate on fitness at a specific level of inbreeding (e.g. Day et al. 2003; Swindell and Bouzat 2006; Kristensen et al. 2011), very few studies have combined these two variables to explore the effect of inbreeding rate on fitness over a range of inbreeding levels (but see Reed et al. 2003; Pekkala et al. 2012b).

The detrimental effects of inbreeding and genetic drift in small populations can be alleviated by mating between individuals from genetically differentiated populations (hybridization; Hedrick et al. 2011). Heterosis, the increased fitness of hybrid offspring, is generally attributed to the masking of recessive deleterious alleles in heterozygous genotypes, and to restoration of heterozygosity in overdominant loci (Charlesworth and Willis 2009). Isolated populations, however, can also accumulate genetic differences that have detrimental effects upon hybridization, that is, that induce outbreeding depression. In the absence of divergent local adaptation, outbreeding depression can be caused by disruption of co-adapted gene complexes (Templeton 1986; Lynch 1991), or by formation of deleterious multilocus interactions (Orr and Turelli 2001; Presgraves 2010). One of the key factors predicted to influence the outcome of hybridization is the level of population divergence (e.g. Lynch 1991; Falconer and Mackay 1996; Orr and Turelli 2001). In the absence of selection, heterosis should increase linearly with population divergence (Falconer and Mackay 1996), whereas outbreeding depression due to multilocus interactions should develop slowly in the beginning, but at accelerated speed as populations become increasingly differentiated (Orr and Turelli 2001). Consistent with the expectation, many empirical studies have found an intermediate optimum or a negative relationship between parental divergence and offspring fitness (reviewed in Edmands 2002, 2007). Most studies, however, have focused on geographically separated natural populations, making it difficult to disentangle the consequences of local adaptation from processes independent of divergent selection pressures in contributing to heterosis and outbreeding depression.

Another factor that can influence the outcome of interpopulation hybridization is inbreeding rate. Populations with slow inbreeding can be expected to possess less potential for heterosis because of stronger purging of recessive deleterious alleles (Wang et al. 1999; Whitlock et al. 2000; Theodorou and Couvet 2006). Previous studies have reported higher heterosis in smaller compared with larger populations (e.g. Paland and Schmid 2003; Willi et al. 2007; Escobar et al. 2008), but we do not know of any that have examined how the rate of inbreeding, independent of inbreeding level, affects the consequences of interpopulation hybridization.

The aim of our study was to determine the effect of inbreeding rate on population mean fitness, on within-population inbreeding depression and on heterosis over a wide range of inbreeding coefficients. The study was conducted with experimental *Drosophila littoralis* (Meigen) populations that were replicated in three sizes: *N* = 2, 10 and 40 (inbreeding rate was highest in the *N* = 2 and lowest in the *N* = 40 populations). The populations were maintained simultaneously with an outbred control population (*N* = 500). From controlled within- and between-population crosses, fitness was assessed from first-generation offspring as egg-to-adult survival and female fecundity.

## Materials and methods

### Study populations

The laboratory population of the boreal drosophilid *D. littoralis* was established with flies collected from a natural population in central Finland (see Pekkala et al. 2012b for details on population establishment and maintenance). To manipulate inbreeding rate, experimental populations were established from this large laboratory population in three different sizes: one breeding pair (N2; 96 replicates), five breeding pairs (N10; 16 replicates) and 20 breeding pairs (N40; 12 replicates). An outbred control population was established with 250 pairs. The increase in the level of inbreeding in the control population was negligible during the experiment (Pekkala et al. 2012b).

The N10, N40 and control populations were established using flies from the seventh generation of the original laboratory population (from here on referred to as generation 0). The populations were established and maintained at the same density of five pairs per bottle (containing 50 mL of malt medium), with constant population size and nonoverlapping generations. Each generation the sexually mature parental flies were allowed to mate and lay eggs in the bottles for 5 days, after which the parental flies were removed. To avoid causing selection on fast egg-to-adult development, the first eclosing offspring were discarded. Seven days later, the offspring were collected and separated according to sex under CO_2_ anaesthesia. The males of *D. littoralis* mature at the earliest 10 days after eclosion (based on Pitnick et al. 1995 and our personal observation). Therefore, when collected 0–7 days after eclosion (as we did), the offspring are expected to be virgin. The collected offspring were kept in plastic vials (8 mL of malt medium) in single-sex groups at a maximum density of 10 flies per vial, and changed to fresh vials every 7 days. When mature, the parental flies for each replicate population were randomly picked among the respective offspring. The offspring not used as parents of the next generation were used for the experimental crosses.

The N2 populations were established five generations later with randomly chosen pairs from the control population (for the N2 populations, this generation is referred to as generation 0). Each generation the parental pairs were allowed to mate and lay eggs for 10 days in plastic vials containing 8 mL of malt medium. To prevent crowding of the larvae (see Pekkala et al. 2011), the pairs were transferred to new vials first after 4 days and then every second day. The procedure for collecting the parental flies for the next generation and the flies for the experimental crosses was the same as described above for the larger population sizes.

### Experimental crosses

We used offspring from the experimental populations (N2, N10 and N40) and the control population for controlled crosses within and between the populations. All cross types (see below) were carried out at several generations following the establishment of the populations, that is, at several levels of inbreeding. We aimed to time the crosses so that we could compare the differently sized populations at the same, or at very similar, inbreeding coefficients. However, as the effective population sizes turned out to be smaller than expected (see Estimation of inbreeding coefficients), the estimated inbreeding coefficients (*f*) varied between the differently sized populations at the generations when crosses were performed (see Table1).

**Table 1:** The number of replicate populations (for heterosis, the number of population pairs) used to estimate population mean fitness, inbreeding depression and heterosis for each combination of population size and generation.

Pop size	Gen	*f*	Number of populations (for heterosis, number of population pairs)								
			Population mean fitness			Inbreeding depression			Heterosis		
			EAS	OF	TF	EAS	OF	TF	EAS	OF	TF
2	1	0.26	76	51	54	–	–	–	–	–	–
2	2	0.38	50	31	35	–	–	–	22	8	8
2	3	0.51	29	11	17	–	–	–	13	3	4
2	4	0.60	17	9	16	–	–	–	7	2	6
2	5	0.68	7	4	7	–	–	–	2	–	2
2	6	0.74	4	3	4	–	–	–	–	–	–
10	3	0.17	16	15	15	–	–	–	8	7	7
10	5	0.26	16	–	–	–	–	–	–	–	–
10	6	0.30	16	15	16	16	16	16	8	8	8
10	7	0.34	15	14	15	–	–	–	7	7	7
10	9	0.42	12	–	–	–	–	–	–	–	–
10	10	0.45	14	14	14	11	11	11	7	6	6
10	13	0.54	11	–	–	–	–	–	–	–	–
10	14	0.57	12	12	12	11	11	11	5	5	5
10	19	0.68	8	–	–	–	–	–	–	–	–
10	20	0.70	8	8	8	8	8	8	4	4	4
40	11	0.21	11	–	–	–	–	–	–	–	–
40	12	0.23	11	11	11	11	11	11	6	6	6
40	19	0.33	10	–	–	–	–	–	–	–	–
40	20	0.35	10	10	10	10	10	10	5	5	5
40	22	0.38	10	–	–	–	–	–	–	–	–
40	23	0.39	10	10	10	10	10	10	5	5	5

Pop size, population size treatment; Gen, parental generation; *f*, estimated inbreeding coefficient of the offspring generation; EAS, egg-to-adult survival; OF, offspring fecundity; TF, total fitness.

#### Random crosses within populations

Random crosses within the populations were carried out using randomly picked males and females from the same replicate population. In the N2 populations, one cross per population was carried out each generation (except at generation 6, when 2–3 crosses per population were carried out due to low number of extant populations). In the N10 and N40 populations, up to eight crosses per population were carried out each generation. In the control population, a minimum of 33 and a maximum of 96 crosses were carried out each generation.

#### Full-sib crosses

Full-sib crosses were carried out within the N10 and N40 populations using offspring from the random crosses as parents (see above). One male and one female offspring from up to six families of each replicate population were randomly paired. Note that for the N2 populations, the random crosses are equal to full-sib crosses.

#### Interpopulation crosses

Interpopulation crosses were carried out between randomly picked males from one replicate population and randomly picked females from another replicate population of the same size. For the N2 populations, four crosses (two reciprocal crosses) per population pair were carried out each generation. Because of the high rate of extinctions and low offspring production in the N2 populations, the population pairs for the crosses were chosen randomly each generation. For the N10 and N40 populations, six crosses (three reciprocal crosses) per population pair were carried out each generation. Population pairs were chosen randomly for the first interpopulation crosses; in subsequent generations, the same population pairs were used, except when not possible due to extinctions or low offspring production. Each replicate population was used for only one population pair within a generation, except on two occasions, when a replicate population was used for two population pairs because of an odd number of replicate populations available. See Table S1 for detailed information on population pairs.

### Fitness assays

#### General procedure for crossing the flies

For all experimental crosses, the parental male and female were placed in a plastic vial (8 mL of malt medium) when mature (age 13–26 days from eclosion). Each pair was transferred to a new vial first after 4 days and then every second day for a total of 6 days to prevent crowding of the larvae (Pekkala et al. 2011) and to facilitate the counting of the eggs. The first 4 days were considered as a familiarization period and were not used for the fitness measurements. From the subsequent 6-day period (a total of three vials), we measured egg-to-adult survival of the offspring and fecundity of the female offspring. A product of the two was used as a proxy for total fitness.

#### Egg-to-adult survival

The number of eggs in each vial was counted under a stereomicroscope. The adult offspring were counted until new flies no longer eclosed. Egg-to-adult survival was calculated as the ratio of the number of adult offspring to the number of eggs over the 6-day period (or, on rare occasions, over 4- or 2-day period if eggs or offspring could not be counted from all vials).

#### Offspring fecundity

One female offspring from each experimental cross was paired with a male randomly picked from the control population. The pair was maintained in plastic vials as described above (4 + 2 + 2 + 2 days). From the last 6-day period, the number of eggs in each vial was counted. Offspring fecundity was measured as the average number of eggs laid in a vial (usually the average of three vials, on rare occasions, the average of two vials or the number of eggs in one vial).

#### Total fitness

Total fitness of the offspring was estimated as a product of the two fitness measures, calculated by multiplying the egg-to-adult survival of the offspring with fecundity of the female offspring. If there were no adult offspring (zero egg-to-adult survival), total fitness was scored as 0.

### Estimation of inbreeding coefficients

We estimated the effective population sizes (*N*_*e*_) of the study populations by analysing variation at eight nuclear microsatellite loci as described in Pekkala et al. (2012b). The estimated *N*_*e*_ was 1.9 for the N2 populations, 8.1 for the N10 populations, 23.2 for the N40 populations and 342 for the control population [see Table S2 and Pekkala et al. (2012b) for details of the analyses]. The inbreeding coefficients (the increase in homozygosity due to finite population size, *f*) for each population size at given generations were calculated using the following equation (Crow and Kimura 1970, p. 102), replacing *N* with the estimated *N*_*e*_, and assuming that the parental flies at generation 0 were not related:
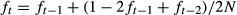


As the replicate populations originate from the same population, the inbreeding coefficient is equal to the level of divergence in allele frequencies (*F*_ST_) between populations of the same size (assuming random mating; Hartl and Clark 1997). In the statistical analyses, we used estimated inbreeding coefficients corresponding to the offspring generation of the experimental crosses (see Table1), because the fitness was measured from the offspring and not from the parental generations.

### Statistical analyses

#### Estimates of population mean fitness

Population mean fitness was estimated relative to the fitness of the control population, as the fitness of the offspring from the random crosses within the experimental populations (N2, N10 and N40) relative to the fitness of the offspring from the random crosses within the control population, measured at the same generation. To calculate the estimate and confidence intervals for each available combination of population size and generation, we first calculated the population-specific means for each fitness measure (egg-to-adult survival, offspring fecundity and total fitness). Mean fitness for each population was then calculated as the logarithm of the ratio of the population mean to the mean of the control population. Next, the estimate of mean fitness for a given population size at each generation was obtained by averaging the population-specific estimates. The confidence intervals were obtained as the parametric 95% confidence limits of the estimate.

To be able to take logarithms from estimates that were zero, we added 0.01 to all population-specific estimates of egg-to-adult survival and 1 to all population-specific estimates of offspring fecundity and total fitness. This procedure was followed also in estimation of inbreeding depression and heterosis (see below). In Figs3, the estimates and confidence intervals have been back-transformed from the logarithmic scale. The number of replicate populations (or, in case of heterosis, the number of population pairs) used for estimating the different variables for each combination of population size and generation is listed in Table1.

**Figure 1:**
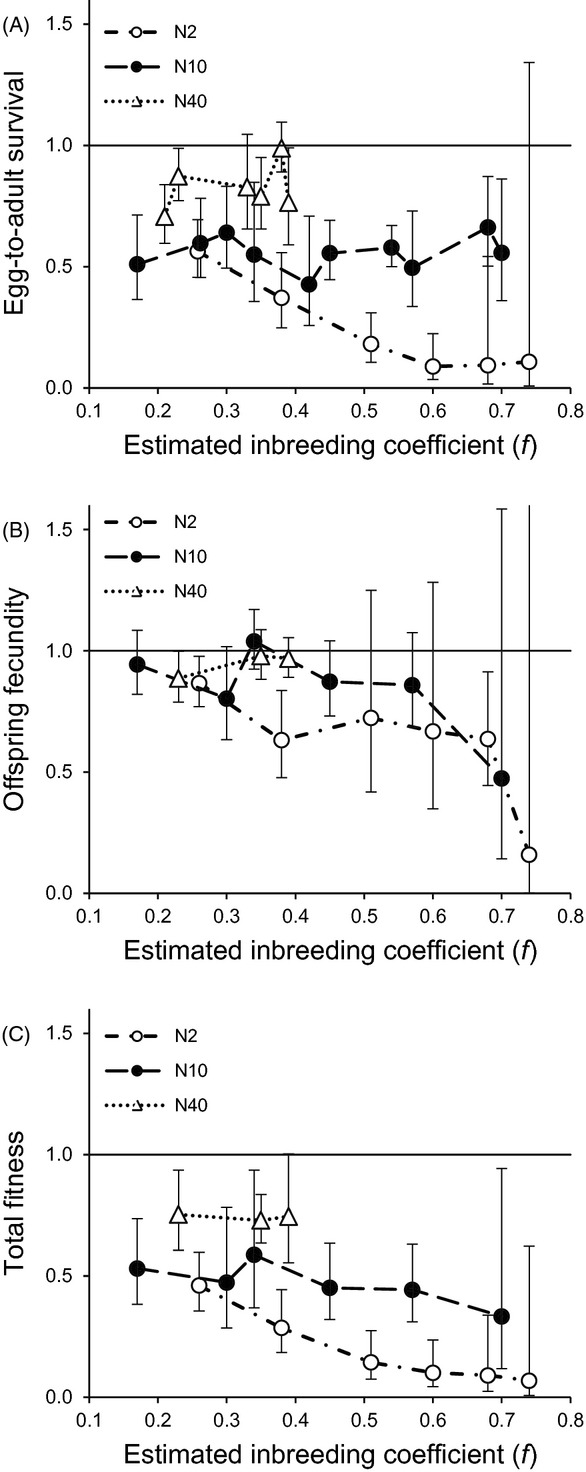
Population mean fitness. Offspring fitness (A: egg-to-adult survival, B: offspring fecundity, C: total fitness) from random crosses within the experimental populations (N2, N10, N40), relative to offspring fitness from random crosses within the control population. The values are means with 95% CI, plotted against estimated inbreeding coefficient (*f*) of the experimental populations. Values <1 indicate reduced fitness relative to the control population (for ease of interpretation, value 1.0 is indicated with solid line).

**Figure 2:**
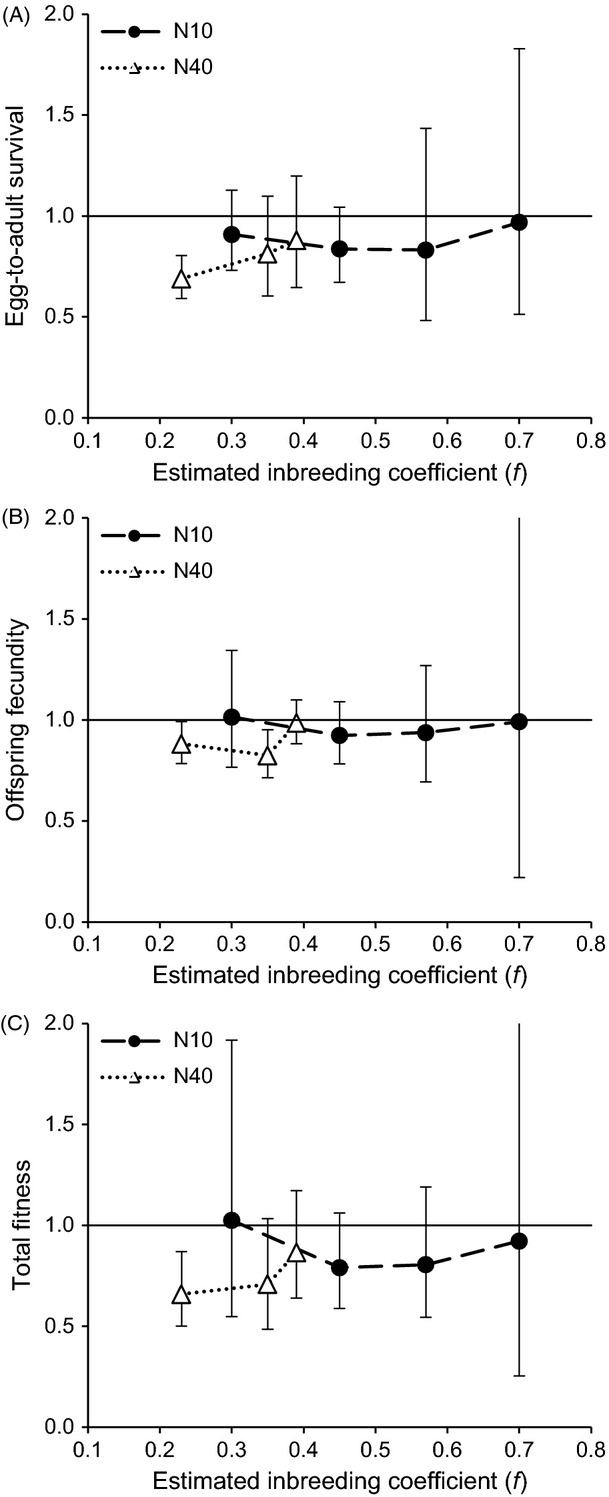
Inbreeding depression. Offspring fitness (A: egg-to-adult survival, B: offspring fecundity, C: total fitness) from full-sib crosses within the experimental populations (N2, N10, N40), relative to offspring fitness from random crosses within the same populations. The values are means with 95% CI, plotted against estimated inbreeding coefficient (*f*) of the experimental populations. Values <1 indicate inbreeding depression (for ease of interpretation, value 1.0 is indicated with solid line).

**Figure 3:**
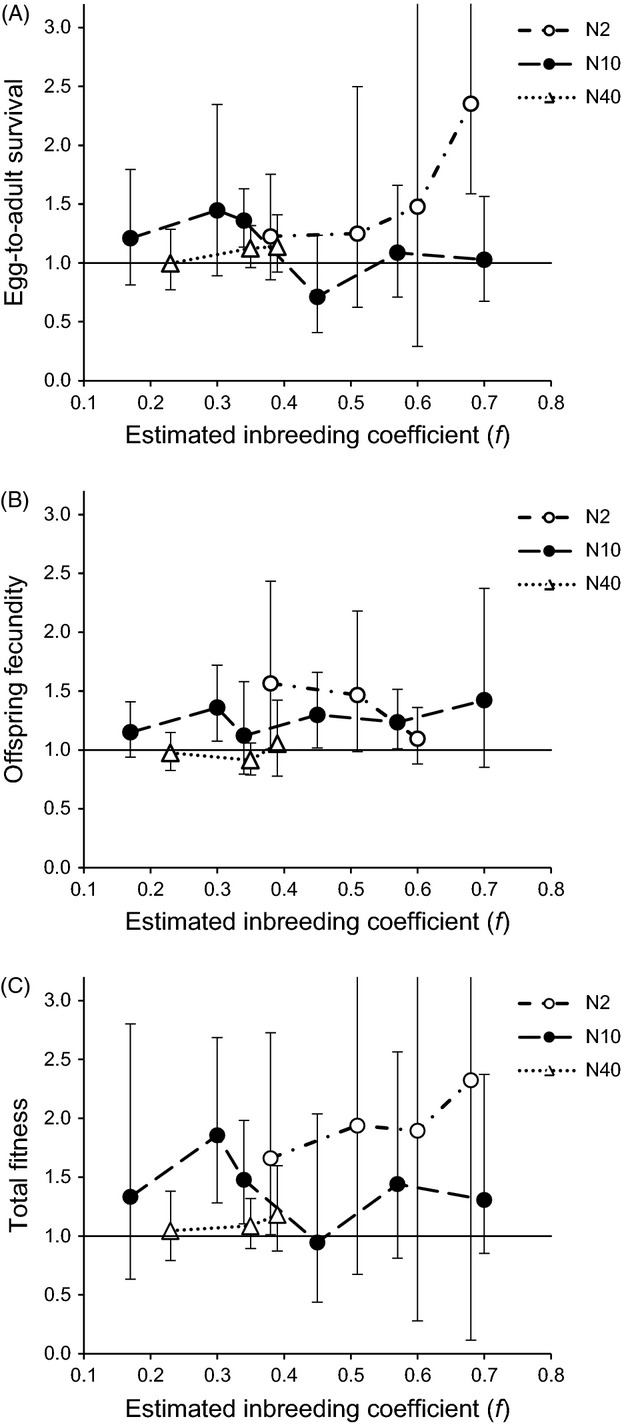
Heterosis. Offspring fitness (A: egg-to-adult survival, B: offspring fecundity, C: total fitness) from crosses between the experimental populations (N2, N10, N40), relative to the mean of offspring fitness from the respective within-population random crosses. The values are means with 95% CI, plotted against estimated inbreeding coefficient (*f*) of the experimental populations. Values >1 indicate heterosis (for ease of interpretation, value 1.0 is indicated with solid line).

#### Estimates of inbreeding depression

Inbreeding depression in the N10 and N40 populations was estimated as the fitness of the offspring from the full-sib crosses relative to the fitness of the offspring from the random crosses within the same population, measured at the same generation. For ease of interpretation, we score and plot inbreeding depression as
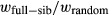


where *w* = fitness, rather than the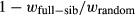 which is often used. This is carried out only to express inbreeding depression in a more intuitive scale (values <1 indicate inbreeding depression), and the choice does not influence the analysis or interpretation of the results.

The estimates of inbreeding depression and their confidence intervals for each available combination of population size and generation were obtained by an approach analogous to that used for the estimates of population mean fitness. First, we calculated the means for each fitness measure (egg-to-adult survival, offspring fecundity and total fitness) for the full-sib crosses and random crosses in each available population for each generation. Inbreeding depression for each population was then calculated as the logarithm of the ratio of the mean of full-sib crosses to the mean of random crosses, measured at the same generation. Next, the estimate of inbreeding depression for a given population size and generation was obtained by averaging the population-specific estimates, and the confidence intervals were obtained as the parametric 95% confidence limits of the estimate.

#### Estimates of heterosis

The magnitude of heterosis was estimated as the fitness of the offspring from the interpopulation crosses relative to the mean of the respective within-population random crosses. Values above 1 thus indicate heterosis, whereas values <1 indicate outbreeding depression. We first calculated the means for each fitness measure (egg-to-adult survival, offspring fecundity and total fitness) for each interpopulation cross and for the random crosses. Heterosis was then calculated as the logarithm of the ratio of the mean of the interpopulation crosses to the mean of the random crosses. Next, the estimate for a given population size and generation was obtained by averaging the estimates of heterosis, and the confidence intervals were obtained as the parametric 95% confidence limits of the estimated average.

#### Statistical models

The effects of population size (i.e. inbreeding rate) and inbreeding level on population mean fitness and inbreeding depression were analysed with a linear mixed model, with population size and inbreeding coefficient (nested within population size) as fixed factors, and the population as a random factor. In both models (for population mean fitness and inbreeding depression), variance among populations was assumed to be equal in different population sizes and at different inbreeding coefficients. In principle, it would also be possible to estimate variance parameters separately for each population size and inbreeding level combination. However, given the structure and amount of the data, models with homogenous variance were considered most appropriate.

The effects of population size and inbreeding level on heterosis were analysed with a linear model with population size and inbreeding coefficient (nested within population size) as fixed factors. We did not identify population as a random factor in this analysis because, due to extinctions or low offspring production, the same population pairs could not always be used in the crosses.

Because we did not have data from all population sizes at similar inbreeding coefficients, in addition to full analyses, we ran analyses with limited inbreeding coefficient ranges to address specific questions. For population mean fitness, separate linear mixed models were built for *f *<* *0.40 to make pairwise comparisons between all three population sizes and for *f *>* *0.40 to compare the N2 and N10 populations. For inbreeding depression, a separate linear mixed model was built for *f *=* *0.30–0.45 to compare the two population sizes (N10 and N40). For heterosis, separate linear models were built for *f *<* *0.40 to compare the N10 and N40 populations, and for *f *=* *0.34–0.60 (in offspring fecundity) or for *f *=* *0.34–0.70 (in egg-to-adult survival and in total fitness) to compare the N2 and N10 populations.

## Results

### Population mean fitness

#### Egg-to-adult survival

In the N2 populations, egg-to-adult survival was lower than in the control population at all but one inbreeding coefficients (judged from the confidence intervals; mean value <1, CI not overlapping 1; Fig.1A; Table S3). In the N10 populations, egg-to-adult survival was lower than in the control population at all measured inbreeding coefficients, and in the N40 populations at four of six inbreeding coefficients (at *f *=* *0.21, *f *=* *0.23, *f *=* *0.35 and *f *=* *0.39).

Egg-to-adult survival (estimated relative to the control population) was affected by both population size and inbreeding level (Table2). At low levels of inbreeding (*f *<* *0.40), survival was lower in the N2 compared with the N40 populations; survival in the N10 populations was not different from the other population sizes (Fig.1A; Table S4). At high levels of inbreeding (*f *>* *0.40), survival was lower in the N2 than in the N10 populations (no data for the N40 populations; Fig.1A; Table S4). In the N2 populations, egg-to-adult survival decreased with increasing level of inbreeding; in the two larger population sizes, inbreeding level had no significant effect (Fig.1A; Table S5).

**Table 2:** Mixed model analysis for population mean fitness (estimated relative to the control population).

Fitness measure	Effect	Num. DF	Den. DF	*F*	*P*
Egg-to-adult survival	Population size	2	38.5	39.80	**<0.001**
	*f* (within population size)	19	225	6.20	**<0.001**
Offspring fecundity	Population size	2	75.5	9.00	**<0.001**
	*f* (within population size)	12	169	3.45	**<0.001**
Total fitness	Population size	2	44.9	33.01	**<0.001**
	*f* (within population size)	12	156	5.38	**<0.001**

*f *= estimated inbreeding coefficient, significant *P*-values (*P* < 0.05) in bold.

#### Offspring fecundity

In the N2 populations, offspring fecundity was lower than in the control population at three of six inbreeding coefficients (at *f *=* *0.26, *f *=* *0.38 and *f *=* *0.68; mean value <1, CI not overlapping 1; Fig.1B; Table S3). The N10 populations did not show evidence for reduced offspring fecundity. In the N40 populations, reduction in offspring fecundity was significant at one of three inbreeding coefficients (*f *=* *0.23).

Offspring fecundity (estimated relative to the control population) was affected by both population size and inbreeding level (Table2). At low levels of inbreeding (*f *<* *0.40), the N2 populations had lower fecundity than the N10 or N40 populations; the N10 and N40 populations did not differ from each other (Fig.1B; Table S4). At high levels of inbreeding (*f *>* *0.40), no effect of population size was detected (data from the N2 and N10 populations only; Fig.1B; Table S4). In the N2 populations, offspring fecundity decreased with increasing level of inbreeding; inbreeding level had no effect on offspring fecundity in the N10 and N40 populations (Fig.1B; Table S5).

#### Total fitness

In the N2 and the N10 populations, total fitness was lower than in the control population at all measured inbreeding coefficients (mean value <1, CI not overlapping 1; Fig.1C; Table S3). In the N40 populations, total fitness was lower than in the control population at two of three inbreeding coefficients (*f *=* *0.23 and *f *=* *0.35).

Both population size and inbreeding level had a highly significant effect on total fitness (estimated relative to the control population; Table2). At low levels of inbreeding (*f *<* *0.40), total fitness was lower in the N2 than in the N10 or N40 populations; the N10 and N40 populations did not differ from each other (Fig.1C; Table S4). At high levels of inbreeding (*f *>* *0.40), total fitness was lower in the N2 than in the N10 populations (no data for the N40 populations; Fig.1C; Table S4). Total fitness decreased with increasing inbreeding level in the N2 populations, but inbreeding level had no effect on total fitness in the N10 or N40 populations (Fig.1C; Table S5).

### Inbreeding depression

The estimates of within-population inbreeding depression for the N10 populations were nonsignificant at all measured inbreeding coefficients. The N40 populations showed inbreeding depression in egg-to-adult survival and in total fitness at one (*f *=* *0.23), and in offspring fecundity at two (*f *=* *0.23 and *f *=* *0.35) of three inbreeding coefficients (mean value <1, CI not overlapping 1; Fig.2; Table S3). The mixed model analysis did not detect significant effects of population size or inbreeding level on inbreeding depression in any of the fitness measures (Tables3, S4 and S5).

**Table 3:** Mixed model analysis for inbreeding depression.

Fitness measure	Effect	Num. DF	Den. DF	*F*	*P*
Egg-to-adult survival	Population size	1	70	0.89	0.348
	*f* (within population size)	5	70	0.37	0.866
Offspring fecundity	Population size	1	68	0.25	0.621
	*f* (within population size)	5	68	0.11	0.990
Total fitness	Population size	1	70	0.80	0.376
	*f* (within population size)	5	70	0.29	0.916

*f *= estimated inbreeding coefficient.

### Heterosis

#### Egg-to-adult survival

The N2 populations showed heterosis in egg-to-adult survival at one of four, and the N10 populations at one of six inbreeding coefficients (at *f *=* *0.68 and at *f *=* *0.34, respectively; mean value >1, CI not overlapping 1; Fig.3A; Table S3). Hybridization had no effect on egg-to-adult survival in the N40 populations. The linear model analysis revealed no effects of population size or inbreeding level on heterosis in egg-to-adult survival (Tables4, S4 and S5).

**Table 4:** Linear model analysis for heterosis.

Fitness measure	Effect	Num. DF	Den. DF	*F*	*P*
Egg-to-adult survival	Population size	2	86	1.19	0.309
	*f* (within population size)	10	86	0.51	0.879
Offspring fecundity	Population size	2	54	5.02	**0.010**
	*f* (within population size)	9	54	0.65	0.750
Total fitness	Population size	2	60	2.46	0.094
	*f* (within population size)	10	60	0.36	0.959

*f *= estimated inbreeding coefficient, significant *P*-values (*P* < 0.05) in bold.

#### Offspring fecundity

In offspring fecundity, the N2 populations showed heterosis at one of three (*f *=* *0.38), and the N10 populations at three of six inbreeding coefficients (*f *=* *0.30, *f *=* *0.45 and *f *=* *0.57; mean value >1, CI not overlapping 1; Fig.3B; Table S3). Hybridization had no effect on offspring fecundity in the N40 populations.

The magnitude of heterosis in offspring fecundity was affected by population size, but not by inbreeding level (Tables4, S4 and S5). At low levels of inbreeding (*f *<* *0.40), heterosis was higher in the N10 than in the N40 populations (only N10 and N40 populations compared; Fig.3B; Table S4). At high levels of inbreeding (*f *=* *0.34–0.60), population size had no significant effect (only N2 and N10 populations compared; Fig.3B; Table S4).

#### Total fitness

The N2 populations showed heterosis in total fitness at one of four inbreeding coefficients (*f *=* *0.38; mean value >1, CI not overlapping 1; Fig.3C; Table S3). Notably, the estimates of mean at the nonsignificant data points were also very high (≥1.89); the nonsignificance of the values was due to the wide confidence intervals resulting from limited sample sizes at high inbreeding levels (see Table1). The N10 populations showed heterosis in total fitness at two of six inbreeding coefficients (*f *=* *0.30 and *f *=* *0.34). Hybridization had no effect on total fitness in the N40 populations.

The linear model analysis did not detect significant effects of population size or inbreeding level on heterosis in total fitness, when all inbreeding coefficients were included in the model (Tables4 and S5). However, at low levels of inbreeding (*f *<* *0.40), heterosis was significantly higher in the N10 than in the N40 populations (only N10 and N40 populations compared; Fig.3C; Table S4). At high levels of inbreeding (*f *=* *0.34–0.70), population size had no effect (only N2 and N10 populations compared; Fig.3C; Table S4).

## Discussion

### Population mean fitness

We found reduced fitness in all three population size treatments (N2, N10 and N40), when compared to the control population. In the N2 and N40 populations, fitness was reduced in all three fitness measures (egg-to-adult survival, offspring fecundity and total fitness); in the N10 populations, egg-to-adult survival and total fitness were reduced. The fitness reduction was likely caused by increased homozygosity of deleterious recessive or partially recessive alleles resulting from increased levels of inbreeding and drift, but could also follow from loss of heterozygosity in overdominant loci, and from formation of deleterious epistatic interactions (Lynch et al. 1995a; Whitlock et al. 2000; Charlesworth and Willis 2009; Edmands et al. 2009). Compared over similar levels of inbreeding, the reduction in fitness was greater in populations with fast inbreeding (greater reduction in the N2, compared with the N10 and N40 populations). Greater reduction in fitness with fast inbreeding suggests that selection against deleterious alleles and allele combinations, and possibly also for maintenance of heterozygosity in overdominant loci, was less efficient in populations of small effective size, as predicted by theoretical models (Wang et al. 1999; Theodorou and Couvet 2006).

Previous empirical studies on the effect of inbreeding rate on fitness have shown inconsistent results: some of them find support for the ‘slower is better’ hypothesis (Ehiobu et al. 1989; Day et al. 2003; Reed et al. 2003; Pedersen et al. 2005; Demontis et al. 2009), whereas others do not find a consistent effect (Mikkelsen et al. 2010; Kristensen et al. 2011). Other studies have shown inbreeding rate to be important only under stressful conditions (Bijlsma et al. 2000; Swindell and Bouzat 2006). These conflicting results suggest that the effect of inbreeding rate on population mean fitness may be influenced by factors such as population demographic history or the study methods used (Crnokrak and Barrett 2002).

Our study differs from most previous studies in that we studied the effect of inbreeding rate over a wide range of inbreeding coefficients (*f *=* *0.17–0.74) rather than focusing on a specific level of inbreeding. The N2 populations showed greater reduction in fitness compared with the larger populations over the whole range of inbreeding coefficients included in the comparison. Moreover, the mean fitness in the N2 populations decreased with increasing level of inbreeding, but no temporal effect was found in the larger populations (note, however, that we did not have data from the N40 populations at the highest inbreeding coefficients). The decrease in fitness in the N2 populations with increasing inbreeding level shows the accumulating effect of inbreeding and drift with proceeding generations (Lande 1994; Lynch et al. 1995a,b; Wang et al. 1999) and indicates that selection is inefficient against the harmful effects of inbreeding and drift when inbreeding is very fast. With increasing homozygosity, deleterious alleles and allele combinations could be eliminated not only through within-population selection, but also through selective loss of most unfit populations. The decreasing fitness of the N2 populations despite the loss of the vast majority (approximately 96%) of the populations during the experiment indicates that among-population selection was not effective in these populations. Our results are consistent with a study where Reed et al. (2003) followed the fitness of *Drosophila melanogaster* populations that differed in inbreeding rate over a range of inbreeding coefficients and found that the decrease in population survival with increasing level of inbreeding was faster in populations with fast inbreeding.

Interestingly, in a previous study where we measured offspring production in the N10 and N40 populations (Pekkala et al. 2012b), we found inbreeding level to have an overall effect on population fitness. However, the effect was not simple. In the N10 populations, a decrease in offspring production at low levels of inbreeding was followed by a transient recovery and again a decrease at higher inbreeding levels (Pekkala et al. 2012b). In the N40 populations, no decrease in offspring production was observed until higher inbreeding levels (*f *>* *0.35) were reached (Pekkala et al. 2012b). The differences in the results of the two studies are most probably caused by differences in the fitness measures; in the previous study, we counted the number of eclosed offspring per population, whereas in the current study we used measures of individual fitness. Both studies, however, support the same conclusion made about the effect of inbreeding rate: faster inbreeding has more severe effects on fitness.

In the current study, the reduction in mean fitness of the populations was greater in egg-to-adult survival than in fecundity of the female offspring. This suggests that the deleterious effects of inbreeding and drift were mainly caused by lethal alleles or allele combinations. However, it has to be recognized that egg-to-adult survival incorporates information from all individuals in the population, while fecundity can only be measured for the selected group that survived from egg to adult. Stronger effects of inbreeding and drift on traits expressed early in life, as compared to traits expressed later in life, have been found also in other studies (see e.g. Husband and Schemske 1996; Saccheri et al. 1996; Escobar et al. 2008).

### Inbreeding depression

We found significant within-population inbreeding depression in the larger (N40), but not in the smaller (N10) populations. One possible explanation for finding inbreeding depression only in the larger populations is that despite similar levels of inbreeding at neutral loci, the larger populations were in fact heterozygous in more loci under selection (Wang et al. 1999; Kristensen et al. 2005; Demontis et al. 2009). However, because of the limited data available for comparison of the two population size treatments, and because the overall difference between the two treatments was not significant, it is not possible to make reliable conclusions about the effects of inbreeding rate on the magnitude of inbreeding depression. Inbreeding depression in the N40 populations was mainly observed at a relatively low level of inbreeding; at *f *=* *0.23, the estimate of inbreeding depression was significant for all fitness measures. Lack of polymorphism resulting from drift and purging of deleterious alleles is a plausible explanation for not finding inbreeding depression at high inbreeding coefficients (Wang et al. 1999; Theodorou and Couvet 2006).

### Heterosis

Hybridization between isolated populations increased fitness of the offspring, that is, induced heterosis, in all three fitness measures. Compared over similar levels of inbreeding, heterosis was higher in populations experiencing faster inbreeding. In fact, significant heterosis was found only in the two smallest (N2 and N10), and not in the largest populations (N40). Finding more heterosis in populations where inbreeding is fast is not surprising, given that also the mean fitness was lower in populations with fast inbreeding. This result gives further support for the inference of more effective selection with slow inbreeding: if larger populations have more efficiently purged deleterious alleles, or maintained diversity in overdominant loci, there simply is not that much to gain from hybridization (Wang et al. 1999; Whitlock et al. 2000; Theodorou and Couvet 2006). Some previous studies have found higher heterosis in smaller compared with larger populations (e.g. Paland and Schmid 2003; Willi et al. 2007; Escobar et al. 2008). However, whether the differences in the magnitude of heterosis detected in these studies result from differences in inbreeding rate remains unknown. To our knowledge, no previous studies exist that have examined the effect of inbreeding rate on the outcome of interpopulation hybridization while controlling for the level of inbreeding in the populations.

The inbreeding coefficient, which equals the level of genetic divergence between the populations, had no overall effect on the magnitude of heterosis. However, the N10 populations expressed significant heterosis in total fitness at intermediate (*f *=* *0.30 and *f *=* *0.34), but not at low (*f *=* *0.17), or at high levels of inbreeding (*f *=* *0.45–0.70). This is consistent with the results of a previous study, in which we used the same N10 populations to study the long-term effects of hybridization on population fitness: we found significant heterosis in populations crossed at intermediate, but not in populations crossed at high inbreeding level (Pekkala et al. 2012a). Not finding heterosis at low levels of inbreeding is not surprising, because there can be no heterosis without population divergence. Not finding heterosis at high levels of inbreeding is more interesting and could be due to purging of highly deleterious recessive alleles from the isolated populations (Whitlock et al. 2000; Glemin 2003; Charlesworth and Willis 2009). If recessive deleterious alleles do not exist, they cannot depress fitness in the isolated populations, and their effects are not masked in the hybrid offspring (less harmful, only partially recessive alleles may still depress fitness of the populations). Another explanation could be formation of negative epistatic interactions when populations are more diverged; the probability for negative effects of hybridization is expected to increase at higher levels of divergence (Lynch 1991; Falconer and Mackay 1996; Orr and Turelli 2001). Our results are consistent with previous studies that have found an intermediate optimum between parental divergence and offspring fitness (reviewed in Edmands 2002, 2007). In most examples, however, the populations studied could have adapted to different environmental conditions, which is not the case for our populations that originated from the same natural population and were maintained under identical conditions.

Heterosis was found in all three fitness measures (egg-to-adult survival, fecundity and total fitness), but offspring fecundity showed significant heterosis more often than egg-to-adult survival. This is surprising considering that the effects of inbreeding and drift on the mean fitness of the populations were more pronounced in early survival of the offspring. One possible explanation is that the negative effects of inbreeding and drift in egg-to-adult survival were to some extent caused by maternal effects. Maternal genes are known to affect early development in *Drosophila* (Powell 1997), and maternal condition (ageing) affects egg-to-adult survival in *D. littoralis* (Pekkala et al. 2011). As the mothers of the hybrid offspring were from small populations, they are likely to be homozygous for some deleterious alleles, and the negative effects of those alleles could outweigh the positive effects of hybridization on the early development of the offspring. It is also possible that there were more negative effects of hybridization on egg-to-adult survival than on offspring fecundity. Some previous studies have found stronger outbreeding depression in early compared with late life-history traits (e.g. Galloway and Etterson 2005; Peer and Taborsky 2005; Escobar et al. 2008).

## Conclusions

We have reported one of the first experimental studies on the effect of inbreeding rate over a range of inbreeding coefficients, on population mean fitness, on within-population inbreeding depression and on heterosis resulting from interpopulation hybridization. In addition to increasing the general understanding on the effects of inbreeding, genetic drift and hybridization in small populations, the findings have special relevance for the management of captive populations and for conservation of threatened species. Our results show that for similar levels of inbreeding, faster inbreeding causes more severe reduction in population mean fitness and, correspondingly, increases the benefits gained from hybridization between populations. However, even slow inbreeding can be detrimental: significant reduction in mean fitness was observed in all population size treatments. The magnitude of within-population inbreeding depression could potentially serve as an indicator of the history or genetic quality of populations. However, with our data, we did not find a clear relationship between inbreeding depression and the rate or the level of inbreeding in the populations. More experimental studies with long-term, replicated set-ups are needed to further enhance our understanding about the consequences of reduced population size and mixing of isolated populations, and to enable general predictions and recommendations. Ideally, these studies should be carried out in various environmental conditions and include measures from several components of fitness at both individual and population levels.

## Data archiving statement

Data for this study are available from the Dryad Digital Repository: http://dx.doi.org/10.5061/dryad.10154.

## Supporting Information

Table S1 The population pairs used for the interpopulation crosses.

Table S2 The results with sample sizes from the analysis of variation at eight nuclear microsatellite loci for the N2 populations.

Table S3 The estimates with 95% confidence limits for population mean fitness, inbreeding depression, and heterosis for each population size and level of inbreeding.

Table S4 Results of statistical analysis on the effect of population size (i.e. inbreeding rate) on population mean fitness, inbreeding depression, and heterosis, at limited inbreeding coefficient ranges.

Table S5 Results of statistical analysis on the effect of inbreeding level on population mean fitness, inbreeding depression, and heterosis, in different population size treatments.
