# Supplementary material for: The effect of inbreeding rate on fitness, inbreeding depression and heterosis over a range of inbreeding coefficients
Source: Evol Appl. 2014 Feb 7;7(9):1107–19. doi: 10.1111/eva.12145 (PMC4231599; doi:10.1111/eva.12145)
Supplement: Table S1 — The population pairs used for the interpopulation crosses. [file eva0007-1107-sd1.docx]

Table S1 Details of the 17 microsatellite loci included in this study. The loci were amplified in three PCR multiplex assays for North Sea houting, Baltic houting, and European whitefish (Mplx A) and three differently composed PCR multiplex assays for vendace (Mplx B) following the conditions reported in Præbel et al. (2013). GB = Genbank accession number, Ta = alignment temperature, C = concentration, Fph = fluorophor, Mplx = PCR multiplex assignment.

| **Locus ID (GB)** | **Ta** | **C (µM)** | **Fph** | **Size range** | **Mplx A** | **Mplx B** |
| --- | --- | --- | --- | --- | --- | --- |
| Bwf1^a^ | 57 | 0.250 | PET | 205-229 | I | I |
| Bwf2^a^ | 57 | 0.100 | PET | 154-174 | I | III |
| BFRO018 (AF175252)^b^ | 60 | 0.080 | PET | 189-199 | II | II |
| ClaTet3 (EU311796)^c^ | 57 | 0.125 | PET | 260-344 | I | - |
| ClaTet5 (EU311798)^c^ | 61 | 0.200 | NED | 110-190 | III | - |
| ClaTet6 (EU311799)^c^ | 61 | 0.150 | 6-FAM | 183-315 | III | III |
| ClaTet9 (EU311802)^c^ | 61 | 0.100 | VIC | 128-204 | III | III |
| ClaTet10 (EU311803 ^c^ | 60 | 0.120 | VIC | 154-438 | II | - |
| ClaTet13 (EU311806 ^c^ | 57 | 0.150 | 6-FAM | 214-258 | I | I |
| ClaTet15 (EU311808)^c^ | 61 | 0.100 | PET | 159-175 | III | - |
| ClaTet18 (EU311811)^c^ | 57 | 0.200 | VIC | 275-351 | I | - |
| Cocl-Lav04 (AY453197)^d^ | 57 | 0.075 | 6-FAM | 139-153 | I | I |
| Cocl-Lav06 (AY453199)^d^ | 57 | 0.150 | NED | 124-142 | I | I |
| Cocl-Lav10 (AY453201)^d^ | 57 | 0.050 | NED | 260-286 | I | I |
| Cocl-Lav18 (AY453203)^d^ | 60 | 0.080 | PET | 151-161 | II | - |
| Cocl-Lav27 (AY453207)^d^ | 57 | 0.040 | VIC | 117-187 | I | I |
| Cocl-Lav49 (AY453212)^d^ | 60 | 0.080 | NED | 164-204 | II | II |

^a^Susnik S, Snoj A, Dovc P (1999) Microsatellites in grayling (*Thymallus thymallus*): comparison of two geographically remote populations from the Danubian and Adriatic river basin in Slovenia. Molecular Ecology 8:1756-1758

^b^Patton JC, Gallaway BJ, Fechhelm RG, Cronin MA (1997) Genetic variation of microsatellite and mitochondrial DNA markers in broad whitefish (*Coregonus nasus*) in the Colville and Sagavanirktok rivers in northern Alaska. Canadian Journal of Fisheries and Aquatic Sciences 54:1548-1556

^c^Winkler KA, Weiss S (2008) Eighteen new tetranucleotide microsatellite DNA markers for *Coregonus lavaretus* cloned from an alpine lake population. Molecular Ecology Resources 8:1055-1058

^d^Rogers SM, Marchand MH, Bernatchez L (2004) Isolation, characterization and cross-salmonid amplification of 31 microsatellite loci in the lake whitefish (*Coregonus clupeaformis*, Mitchill). Molecular Ecology Notes 4:89-92
