# Supplementary material for: The effect of inbreeding rate on fitness, inbreeding depression and heterosis over a range of inbreeding coefficients
Source: Evol Appl. 2014 Feb 7;7(9):1107–19. doi: 10.1111/eva.12145 (PMC4231599; doi:10.1111/eva.12145)
Supplement: Table S2 — The results with sample sizes from the analysis of variation at eight nuclear microsatellite loci for the N2 populations. [file eva0007-1107-sd2.docx]

Table S2 Gill raker counts (GRC) for indigenous and reintroduced North Sea houting (NSH), Baltic houting (BH) and European whitefish (EW) populations across the study area, with supplementary literature values for indigenous NSH populations for which no GRC could be obtained in this study.

| Taxon | Population | Year | Status | N | Mean | SD | Median | Range | Source |
| --- | --- | --- | --- | --- | --- | --- | --- | --- | --- |
| **NSH_** | Danish North Sea inflows | 1845-1890 | Indigenous | 10 | 30.0 | 1.9 | - | 28-34 | Schöter (2002) |
|  | TRE | 2009-2011 | Reintroduced | 86 | 31.9 | 2.6 | 32 | 24-39 | This study |
|  | TRE | 2000 | Reintroduced | 19 | 32.0 | 1.7 | - | 28-35 | Schöter (2002) |
|  | ELB | 1860 | Indigenous | 2 | 32.5 | 3.5 | 32.5 | 30-35 | Schöter (2002) |
|  | ELB | 2010 | Reintroduced | 19 | 33.2 | 3.9 | 33 | 27-41 | This study |
|  |  |  |  |  |  |  |  |  |  |
| **BH_** | PEE | 2010-2011 | Indigenous | 24 | 28.6 | 1.8 | 28 | 26-33 | This study |
|  | TRA | 2010-2011 | Reintroduced | 39 | 29.1 | 2.7 | 29 | 25-35 | This study |
|  | NOK | 2010-2012 | Reintroduced | 4 | 37.3 | 3.0 | 37 | 33-42 | This study |
|  | LAC | 2011 | Reintroduced | 5 | 35.0 | 1.8 | 35 | 33-37 | This study |
|  | SCH | 2010-2011 | Reintroduced | 19 | 31.2 | 2.5 | 31 | 26-36 | This study |
|  | SCH | 1918-1919 | - | 16 | 31.0 |  | - | 25-34 | Thienemann (1922) |
|  | SCH | 2000 | Reintroduced | 27 | 28.0 | 3.5 | - | 23-33 | Schöter (2002) |
|  |  |  |  |  |  |  |  |  |  |
| **LW_** | BOR | 2011 | - | 5 | 38.0 | 5.7 | 40 | 29-43 | This study |
|  | POE | 2010 | - | 13 | 41.7 | 2.7 | 41 | 36-48 | This study |

Year, year of catch; Status, either indigenous or reintroduced by stocking after local extinction. SD, standard deviation.
