# Supplementary material for: The effect of inbreeding rate on fitness, inbreeding depression and heterosis over a range of inbreeding coefficients
Source: Evol Appl. 2014 Feb 7;7(9):1107–19. doi: 10.1111/eva.12145 (PMC4231599; doi:10.1111/eva.12145)
Supplement: Table S3 — The estimates with 95% confidence limits for population mean fitness, inbreeding depression, and heterosis for each population size and level of inbreeding. [file eva0007-1107-sd3.docx]

Table S3. Estimates of pairwise genetic differentiation (*F*_st_) between source and reintroduced populations of North Sea houting (NSH), Baltic houting (BH) and European whitefish (EW) based on mtDNA data^a^.

|  | NSH_ |  |  |  | BH_ |  |  |  |  | EW_ |  |  |
| --- | --- | --- | --- | --- | --- | --- | --- | --- | --- | --- | --- | --- |
|  | VID | TRE | RHI | ELB | SCH | LAC | NOK | TRA | PEE | BOR | POE | PIN |
| VID |  | N.S. | N.S | N.S. | *** | *** | *** | *** | *** | *** | *** | *** |
| TRE | 0.02 |  | * | N.S. | *** | * | *** | *** | *** | *** | *** | *** |
| RHI | 0.02 | 0.03 |  | *** | *** | *** | *** | *** | *** | *** | *** | *** |
| ELB | 0.07 | 0.02 | 0.15 |  | N.S. | N.S. | N.S. | *** | *** | *** | *** | *** |
| SCH | 0.18 | 0.09 | 0.22 | 0.00 |  | N.S. | * | *** | *** | * | *** | *** |
| LAC | 0.20 | 0.09 | 0.25 | 0.01 | 0.02 |  | ** | *** | *** | *** | *** | *** |
| NOK | 0.35 | 0.34 | 0.46 | 0.08 | 0.10 | 0.02 |  | *** | *** | N.S. | N.S. | * |
| TRA | 0.56 | 0.43 | 0.60 | 0.30 | 0.20 | 0.22 | 0.15 |  | * | * | N.S. | * |
| PEE | 0.72 | 0.54 | 0.73 | 0.51 | 0.37 | 0.40 | 0.33 | 0.03 |  | *** | *** | N.S. |
| BOR | 0.45 | 0.35 | 0.52 | 0.16 | 0.11 | 0.12 | 0.03 | 0.03 | 0.15 |  | N.S. | N.S. |
| POE | 0.43 | 0.38 | 0.52 | 0.15 | 0.13 | 0.14 | 0.02 | 0.07 | 0.17 | 0.01 |  | N.S. |
| PIN | 0.61 | 0.43 | 0.66 | 0.27 | 0.19 | 0.21 | 0.12 | 0.01 | 0.06 | 0.04 | 0.03 |  |

^a^*F*_st_ values shown below the diagonal, and their significance levels above the diagonal (N.S.: not significant; *: *P* < 0.05, but not significant after correction for multiple tests; **: *P* < 0.01; ***: *P* < 0.001). Colored areas mark comparisons within *a priori* taxon.
