# Supplementary material for: The effect of inbreeding rate on fitness, inbreeding depression and heterosis over a range of inbreeding coefficients
Source: Evol Appl. 2014 Feb 7;7(9):1107–19. doi: 10.1111/eva.12145 (PMC4231599; doi:10.1111/eva.12145)
Supplement: Table S4 — Results of statistical analysis on the effect of population size (i.e. inbreeding rate) on population mean fitness, inbreeding depression, and heterosis, at limited inbreeding coefficient ranges. [file eva0007-1107-sd4.docx]

Table S4. Tests values for recent demographic expansion or bottlenecks based on mtDNA. Tajima’s D, Fu’s F, SSD and the raggedness index r with their corresponding *P*-values. Bold print indicates statistical significance after false discovery rate correction.

| Population | Tajima's *D* | *P*-value | Fu's *Fs* | *P*-value | SSD^a^ | *P*-value | Raggedness (*r*) | *P*-value |
| --- | --- | --- | --- | --- | --- | --- | --- | --- |
| NSH_VID | 0.616 | 0.761 | 3.594 | 0.949 | 0.068 | 0.270 | 0.163 | 0.460 |
| NSH_TRE | 0.250 | 0.708 | 3.389 | 0.889 | 0.116 | 0.070 | 0.243 | 0.250 |
| NSH_ELB | -0.759 | 0.249 | 3.100 | 0.928 | 0.088 | 0.110 | 0.131 | 0.170 |
| NSH_RHI | -0.130 | 0.480 | 2.220 | 0.850 | 0.077 | 0.020 | 0.599 | 0.580 |
| BH_SCH | 0.798 | 0.838 | 1.893 | 0.824 | 0.117 | 0.070 | 0.263 | 0.040 |
| BH_LAC | 0.378 | 0.652 | 1.432 | 0.793 | 0.026 | 0.810 | 0.045 | 0.800 |
| BH_NOK | 2.354 | 0.991 | 4.166 | 0.954 | 0.213 | 0.070 | **0.444** | **0.010** |
| BH_TRA | 0.070 | 0.599 | 2.332 | 0.866 | 0.033 | 0.550 | 0.075 | 0.880 |
| BH_PEE | -0.958 | 0.163 | 0.698 | 0.651 | 0.010 | 0.040 | 0.123 | 0.290 |
| EW_BOR | 0.729 | 0.789 | -0.614 | 0.421 | 0.020 | 0.690 | 0.047 | 0.760 |
| EW_POE | -0.518 | 0.354 | 0.813 | 0.666 | 0.029 | 0.610 | 0.060 | 0.680 |
| EW_PIN | -0.744 | 0.246 | 3.118 | 0.895 | 0.078 | 0.020 | 0.697 | 0.660 |

^a^Sum of squared deviations, based on the model of the mismatch distribution.
