# Supplementary material for: The effect of inbreeding rate on fitness, inbreeding depression and heterosis over a range of inbreeding coefficients
Source: Evol Appl. 2014 Feb 7;7(9):1107–19. doi: 10.1111/eva.12145 (PMC4231599; doi:10.1111/eva.12145)
Supplement: Table S5 — Results of statistical analysis on the effect of inbreeding level on population mean fitness, inbreeding depression, and heterosis, in different population size treatments. [file eva0007-1107-sd5.docx]

Table S5 AMOVA results based on the microsatellite dataset, with *a priori* taxon affiliation (North Sea houting, Baltic houting, European whitefish) and populations as grouping levels.

| Source of variation | d.f. | Sum of Squares | Variance components | % of variation |
| --- | --- | --- | --- | --- |
| Among groups | 2 | 120.9 | 0.22 | 3.68 |
| Among populations within groups | 9 | 140.6 | 0.20 | 3.33 |
| Within populations | 602 | 3366.5 | 5.59 | 92.99 |
| Total | 613 | 3628.1 | 6.01 |  |
|  |  |  |  |  |
| Fixation Indices |  |  |  |  |
| *F*_st_: 0.070 (*P*<0.001) |  |  |  |  |
| *F*_sc_: 0.035 (*P*<0.001) |  |  |  |  |
| *F*_ct_: 0.037 (*P*<0.001) |  |  |  |  |
